# Population‐calibrated multiple imputation for a binary/categorical covariate in categorical regression models

**DOI:** 10.1002/sim.8004

**Published:** 2018-10-16

**Authors:** Tra My Pham, James R Carpenter, Tim P Morris, Angela M Wood, Irene Petersen

**Affiliations:** ^1^ Department of Primary Care and Population Health University College London London UK; ^2^ London Hub for Trials Methodology Research MRC Clinical Trials Unit at UCL London UK; ^3^ Department of Medical Statistics London School of Hygiene and Tropical Medicine London UK; ^4^ Department of Public Health and Primary Care University of Cambridge Cambridge UK

**Keywords:** electronic health records, missing data, missing not at random, multiple imputation, sensitivity analysis

## Abstract

Multiple imputation (MI) has become popular for analyses with missing data in medical research. The standard implementation of MI is based on the assumption of data being missing at random (MAR). However, for missing data generated by missing not at random mechanisms, MI performed assuming MAR might not be satisfactory. For an incomplete variable in a given data set, its corresponding population marginal distribution might also be available in an external data source. We show how this information can be readily utilised in the imputation model to calibrate inference to the population by incorporating an appropriately calculated offset termed the “calibrated‐δ adjustment.” We describe the derivation of this offset from the population distribution of the incomplete variable and show how, in applications, it can be used to closely (and often exactly) match the post‐imputation distribution to the population level. Through analytic and simulation studies, we show that our proposed calibrated‐δ adjustment MI method can give the same inference as standard MI when data are MAR, and can produce more accurate inference under two general missing not at random missingness mechanisms. The method is used to impute missing ethnicity data in a type 2 diabetes prevalence case study using UK primary care electronic health records, where it results in scientifically relevant changes in inference for non‐White ethnic groups compared with standard MI. Calibrated‐δ adjustment MI represents a pragmatic approach for utilising available population‐level information in a sensitivity analysis to explore potential departures from the MAR assumption.

## 1 INTRODUCTION

Multiple imputation (MI)^1^ has increasingly become a popular tool for analyses with missing data in medical research^2^, ^3^; the method is now incorporated in many standard statistical software packages.^4^, ^5^, ^6^ In MI, several completed data sets are created, and in each, missing data are replaced with values drawn from an imputation model, which is the Bayesian posterior predictive distribution of the missing data, given the observed data. Each completed data set is then analysed using the substantive analysis model that would have been used with no missing data. This process generates several sets of parameter estimates, which are then combined into a single set of results using Rubin's rules.^1^, ^7^ Given congenial specification of the imputation model, Rubin's rules provide estimates of standard errors and confidence intervals (CI) that correctly reflect the uncertainty due to missing data.

The standard implementation of MI in widely available software packages provides valid inference under the assumption that missing values are missing completely at random (MCAR) or missing at random (MAR). However, in many applied settings, it is possible that the unseen data are missing not at random (MNAR). For example, in primary care, individuals with more frequent blood pressure readings may, on average, have higher blood pressure compared with the rest of the primary care population. Although MI can be used when data are MNAR, imputation becomes more difficult because a model for the missing data mechanism needs to be specified, which describes how missingness depends on both observed and unobserved quantities. This implies that, in practice, it is necessary to define a model for either the association between the probability of observing a variable and its unseen values (selection models),^8^ or the difference in the distribution of subjects with and without missing data (pattern‐mixture models).^9^, ^10^ Due to the potential complexity of modelling the missingness mechanism under MNAR, analyses assuming MNAR are relatively infrequently performed and reported in the applied literature. Instead, in practice, researchers more often try to enhance the plausibility of the MAR assumption as much as possible by including many variables in the imputation model.^11^, ^12^

The extra model specification requirement in MI for MNAR data raises several issues. First, the underlying MAR and MNAR mechanisms are not verifiable from the observed data alone. Second, there can be an infinite number of possible MNAR models for any data set, and it is very rare to know which of these models is appropriate for the missingness mechanism. However, for an incomplete variable in a given data set, its corresponding population marginal distribution might be available from an external data source, such as a population census or survey. If our study sample in truth comes from such a population, it is sensible to feed this information into the imputation model to calibrate inference to the population.

In this paper, we propose a version of MI for an incomplete binary/categorical covariate in categorical regression models, termed calibrated‐δ adjustment MI, which exploits such external information. In this approach, the population distribution of the incomplete variable can be used to calculate an adjustment in the imputation model's intercept, which is used in MI such that the post‐imputation distribution much more closely (and often exactly) matches the population distribution. The idea of the calibrated‐δ adjustment is motivated by van Buuren et al's δ adjustment (offset) approach in MI.^13^ However, whilst values of δ are often chosen arbitrarily (and independently of covariates in the imputation model) in van Buuren et al's approach, the incomplete variable's population distribution is used to derive the value of δ in calibrated‐δ adjustment MI. We show that our proposed method gives equivalent inference to standard MI when data are MAR, and can produce unbiased inference under two general MNAR mechanisms.

From a practical point of view, the development of calibrated‐δ adjustment MI is motivated by the issue of incomplete recording of ethnicity data in UK primary care electronic health records. Routine recording of ethnicity has been incorporated at the general practice level in the UK, and the variable is therefore available in many large primary care databases. However, research addressing ethnicity has been constrained by the low level of recording.^14^, ^15^, ^16^ Studies often handle missing data in ethnicity by either dropping ethnicity from the analysis,^17^ performing a complete record analysis (CRA) (ie, excluding individuals with missing data), or single imputation of missing values with the White ethnic group^18^; these methods will generally lead to biased estimates of association and standard errors.^2^ In addition, the probability that ethnicity is recorded in primary care may well vary systematically by ethnic groups, even after adjusting for other variables.^16^ This implies a potential MNAR mechanism for ethnicity, and as a result, standard MI might fail to give valid inference for the underlying population. Since the population marginal distribution of ethnicity is available in the UK census data, the plausibility of the MAR assumption for ethnicity in UK primary care data can be assessed by using standard MI to handle missing data and comparing the resulting ethnicity distribution to that in the census. In earlier work, we explored departures from the MAR assumption for several incomplete heath indicators such as height, body weight, and blood pressure, as well as lifestyle factors including smoking status and alcohol consumption, by comparing the results with external nationally representative data sets.^19^, ^20^) As an example of this, Marston et al reported that if smoking status is missing for a patient, then he or she is typically either an ex‐smoker or nonsmoker, and accordingly proposed only allowing imputed data to take one of these two values.^20^ The method we describe here supersedes this ad‐hoc approach, providing a way to incorporate population distribution information into MI.

This paper focuses on missing data in an incomplete binary/categorical covariate in an analysis model where the outcome variable and other covariates are all binary/categorical and fully observed. The remainder of this paper is structured as follows. Section 2 works through an example analytically to describe the derivation of the calibrated‐δ adjustment. In Section 3, we formally introduce the procedure of calibrated‐δ adjustment MI and evaluate the performance of the method in simulation studies. Section 4 illustrates the application of this MI method in a case study using electronic health records to examine the association between ethnicity and the prevalence of type 2 diabetes diagnoses in UK primary care. We conclude this paper with a discussion in Section 5.

## 2 ANALYTIC STUDY: BIAS IN A **2 × 2** CONTINGENCY TABLE

In this section, we present the development of calibrated‐δ adjustment MI in a simple setting of a 2 × 2 contingency table and describe the derivation of the calibrated‐δ adjustment.

### 2.1 Study design

Suppose it is of interest to study the association between a binary variable x taking values j = 0,1 and a binary outcome y taking values k = 0,1, whose full‐data distribution is given in Table 1A. The full‐data distribution is assumed to be identical to the population distribution, such that the population marginal distribution of x is given by $p_{j}^{\text{pop}}=\frac{n_{j+}}{n_{++}}$. The data generating model is

$$\text{logit}\left[p\left(y=1\;|\;x\right)\right]=\beta_{0}+\beta_{x}x,$$

whose parameters can be written in terms of cell counts, $\beta_{0}=\ln\left(\frac{n_{01}}{n_{00}}\right)$ and $\beta_{x}=\ln\left(\frac{n_{11}n_{00}}{n_{01}n_{10}}\right)$.

**Table 1:** Analytic study: distribution of x and y and selection models for missingness in x. r, response indicator of x; j and k, index categories of x and y, respectively; j,k take values 0/1 [Corrections added on 17 December 2018, after first online publication: the equations in this table have been corrected]

(A) Distribution in the full data of size n.				
	y = **0**	y = **1**	$\sum_{k=0}^{1}y=k$	
x = 0	n_00_	n_01_	n_0+_	
x = 1	n_10_	n_11_	n_1+_	
$\sum_{j=0}^{1}x=j$	n_+0_	n_+1_	n_++_	
**(B) Distribution amongst subjects with observed x (y is fully observed).**				
	**y** = **0**|**r** = **1**	**y** = **1**|**r** = **1**	$\sum_{k=0}^{1}y=k|r=1$	**Population**
x = 0|r = 1	$n_{00}^{\text{obs}}$	$n_{01}^{\text{obs}}$	$n_{0+}^{\text{obs}}$	n_0+_
x = 1|r = 1	$n_{10}^{\text{obs}}$	$n_{11}^{\text{obs}}$	$n_{1+}^{\text{obs}}$	n_1+_
$\sum_{j=0}^{1}x=j|r=1$	$n_{+0}^{\text{obs}}$	$n_{+1}^{\text{obs}}$	$n_{++}^{\text{obs}}$	
$\sum_{j=0}^{1}x=j|r=0$	$n_{+0}^{\text{mis}}$	$n_{+1}^{\text{mis}}$	$n_{++}^{\text{mis}}$	
**(C) Selection models for missingness in x.**				
**Linear predictor of selection model**			**Selection probability**	**Label**
$\text{logit}\left[p\left[(r=1|x,y\right)\right]$			$p\left(r_{jk}=1\right)$	
α_0_			p_r_	M1
α_0_ + α_y_ y			$p_{r_{k}}$	M2
α_0_ + α_x_ x			$p_{r_{j}}$	M3
α_0_ + α_x_ x + α_y_ y			$p_{r_{jk}}$	M4

In addition, suppose that y is fully observed, whilst some data in x are set to missing (ie, the sample contains no individuals with missing y and observed x, Table 1B). Let r be the response indicator taking values 1 if x is observed and 0 if x is missing. Four different missingness mechanisms considered for x and the corresponding selection models are presented in Table 1C. Observed cell counts, $n_{jk}^{\text{obs}}$, can be written as a product of the full‐data cell counts, n_jk_, and the cell‐wise probability of observing x, $p_{r_{jk}}$, such that $n_{jk}^{\text{obs}}=n_{jk}p_{r_{jk}}$.

To perform standard MI of missing values in x, an imputation model

(1) $$\text{logit}\left[p\left(x=1\;|\;y\right)\right]=\theta_{0}+\theta_{y}y,$$

is fitted to the $n_{++}^{\text{obs}}$ complete records (Table 1B) to obtain the θ parameter estimates, where

$$\theta_{0}^{\text{obs}}=\ln\left(\frac{n_{10}^{\text{obs}}}{n_{00}^{\text{obs}}}\right);\;\theta_{y}^{\text{obs}}=\ln\left(\frac{n_{11}^{\text{obs}}n_{00}^{\text{obs}}}{n_{01}^{\text{obs}}n_{10}^{\text{obs}}}\right).$$

When x is MCAR or MAR conditional on y (Table 1C, M1, and M2, respectively), we can obtain an unbiased estimate of the association between x and y in the missing data by fitting the aforementioned logistic regression imputation model to the complete records. No adjustment is needed in the intercept of the imputation model, and standard MI provides unbiased estimates of the marginal distribution of x as well as the association between x and y. We focus on two general MNAR mechanisms, in which missingness in x depends either on x or both x and y (Table 1C, M3, and M4, respectively). We show in Web Appendix A1 that, under these two MNAR missingness mechanisms, adjusting the intercept of the imputation model for the covariate x can sufficiently correct bias introduced by missing data in x.

### 2.2 Derivation of the calibrated‐**δ** adjustment

We now demonstrate how the population distribution of x can be used to calculate the correct adjustment in the imputation model's intercept under MNAR missingness mechanisms M3 and M4. This adjustment is referred to as the “calibrated‐δ adjustment” to clarify its relationship with van Buuren et al's δ adjustment.^13^

The probability of x = 1 can be written in terms of the conditional probabilities amongst subjects with observed and missing x

$$p\left(x=1\right)=p\left(x=1\;|\;r=1\right)p\left(r=1\right)+p\left(x=1\;|\;r=0\right)p\left(r=0\right),$$

where $p\left(x=1\right)$ is the population proportion; $p\left(x=1\;|\;r=1\right)$ , $p\left(r=1\right)$, and $p\left(r=0\right)$ can be obtained from the observed data. Thus, $p\left(x=1\;|\;r=0\right)$ can be solved for as

(2) $$p\left(x=1\;|\;r=0\right)=\frac{p\left(x=1\right)-p\left(x=1\;|\;r=1\right)p\left(r=1\right)}{p\left(r=0\right)}.$$

Note that $p\left(x=1\;|\;r=0\right)$ can be further written as

(3) $$\begin{array}{ll} p\left(x=1\;|\;r=0\right) & =\sum_{k=0}^{1}\;p\left(x=1\;|\;y=k,r=0\right)p\left(y=k\;|\;r=0\right)\\ & =\frac{1}{n_{++}^{\text{mis}}}\;\sum_{k=0}^{1}\;\text{expit}\left(\theta_{0}^{\text{mis}}+\theta_{y}^{\text{mis}}I\left[y=k\right]\right)n_{+k}^{\text{mis}}, \end{array}$$

where $I\left[A\right]$ is an indicator function taking values 1 if A is true and 0 otherwise. It is shown in Web Appendix A1 that, when x is MNAR dependent on either the values of x or both x and y, $\theta_{y}^{\text{obs}}=\theta_{y}^{\text{mis}}$; (3) [Corrections added on 17 December 2018, after first online publication: Equation (3) has been corrected] is therefore equal to

$$\begin{array}{ll} p\left(x=1\;|\;r=0\right) & =\frac{1}{n_{++}^{\text{mis}}}\;\sum_{k=0}^{1}\;\text{expit}\left(\theta_{0}^{\text{mis}}+\theta_{y}^{\text{obs}}I\left[y=k\right]\right)n_{+k}^{\text{mis}}\\ & =\frac{1}{n_{++}^{\text{mis}}}\;\sum_{k=0}^{1}\;\text{expit}\left[\left(\theta_{0}^{\text{obs}}+\delta \right)+\theta_{y}^{\text{obs}}I\left[y=k\right]\right]n_{+k}^{\text{mis}}\\ & =\frac{1}{n^{\text{mis}}}\sum_{i=1}^{n^{\text{mis}}}\text{expit}\left[\left(\theta_{0}^{\text{obs}}+\delta \right)+\theta_{y}^{\text{obs}}y_{i}\right], \end{array}$$

where δ is the adjustment factor in the intercept of the imputation model for x [Corrections added on 17 December 2018, after first online publication: the preceding equation has been corrected]. The value of the calibrated‐δ adjustment can be obtained numerically from (2) and (3) using interval bisection^21^, ^22^ (or any other root‐finding method).

When the population marginal distribution of the incomplete covariate x is available, a natural alternative to adjusting the intercept of the imputation model based on this information is to weight the complete records in the imputation model (which we term “weighted MI”) to match the post‐imputation distribution of x to the population. In Web Appendix A2, we explore two such weighting approaches, ie, marginal and conditional weighted MI; we show analytically that, whilst these methods can provide more accurate results compared with standard MI under certain MNAR mechanisms, they do not provide a general solution as does calibrated‐δ adjustment MI.

## 3 SIMULATION STUDIES

This section presents univariate simulation studies to evaluate performance measures of the calibrated‐δ adjustment MI method for an incomplete binary covariate x, when the fully observed outcome variable y is also binary. The term “univariate” is used here to refer to the setting where missingness occurs in a single covariate. The aims of these simulation studies are (i) to examine finite‐sample properties of calibrated‐δ adjustment MI including bias in parameter estimates, efficiency in terms of the empirical and average model standard errors, and coverage of 95% CIs and (ii) to compare the method with standard MI and CRA under various missingness mechanisms for x.

### 3.1 When the population distribution is “known”

We consider the setting where the population distribution of the incomplete variable is obtained from a population census or equivalent, ie, it is “known.” The uncertainty associated with having to estimate the population distribution is explored in Section 3.2.

#### 3.1.1 Method

Similar to the analytic study presented in Section 2, the analysis model in this simulation study is a logistic regression model for a fully observed binary outcome y on an incomplete binary covariate x. Calibrated‐δ adjustment MI is compared with standard MI and CRA under four missingness mechanisms of increase complexity. The data generating mechanism and analysis procedures are as follows.

1. Simulate n = 5000 complete values of the binary 0/1 covariate x and binary 0/1 outcome y from the following models.
  (4) $$\begin{array}{ll} & x∼\text{Bernoulli}\left(p_{x}^{\text{pop}}=0.7\right);\\ & \text{logit}\left[p\left(y=1\;|\;x\right)\right]=\beta_{0}+\beta_{x}x, \end{array}$$
  where β_0_ and β_x_ are arbitrarily set to ln(0.5) and ln(1.5), respectively. The same values of the β parameters are used throughout to make bias comparable across all simulation settings. This sample size is chosen to minimise the issue of small‐sample bias associated with the logistic regression.^23^
2. Simulate a binary indicator of response r of x from each of the selection models M1 to M4 (Table 1C). Values of 1.5 and −1.5 are chosen for α_y_ and α_x_ in M2 and M3, respectively, to reflect strong odds ratios (ORs) of observing x (OR =4.5 and 0.2, respectively). For M4, α_y_ = 1.5 and α_x_ = −1.5 are chosen as bias in the two MI methods under evaluation is likely to be apparent with these coefficients predicting missingness in x. For all selection models, α_0_ is altered to achieve approximately 45% missing x. For M1, α_0_ is calculated directly as $\ln\left(\frac{0.55}{0.45}\right)$; for M2 to M4, α_0_ = −0.2;1.35 and 0.75 appear to work well.
3. For i = 1,…,5000, set x_i_ to missing if r_i_ = 0.
4. Impute missing values in x M = 50 times using standard MI and calibrated‐δ adjustment MI in turn.
5. In each MI method, fit the analysis model (4) to each completed data set and combine the results using Rubin's rules.^1^, ^7^

Steps 1 to 5 are repeated S = 2000 times under each of the four selection models M1 to M4, so the same set of simulated independent data sets is used to compare the two MI methods under the same missingness scenario, but a different set of data sets is generated for each missingness scenario.^24^ The parameters of interest are β_0_ and β_x_; although, in practice, β_x_ is usually of more interest. Bias, efficiency of ${\hat{\beta }}_{0}$ and ${\hat{\beta }}_{x}$ in terms of the empirical standard errors, and coverage of 95% CIs are calculated over 2000 repetitions for each combination of simulation settings,^25^ with analyses of full data (ie, before any values in x are set to missing) and complete records also provided for comparison.

All simulations are performed in Stata 14^26^; mi impute logit is used for standard MI, the community‐contributed command uvis logit 27 for calibrated‐δ adjustment MI, and mi estimate: logit for fitting the analysis model to the completed data sets and combining the results using Rubin's rules.^1^, ^7^ Simulated data sets are analysed using the community‐contributed command simsum.^25^

Based on the analytic calculations presented in Section 2, we propose the following procedure for imputing missing values in the covariate x using calibrated‐δ adjustment MI.

1. Fit a logistic regression imputation model for x conditional on y to the complete records to obtain the maximum likelihood estimates of the imputation models' parameters $\hat{\theta }$ and their asymptotic sampling variance $\hat{U}$.
2. Draw new parameters $\tilde{\theta }$ from the large‐sample normal approximation $N(\hat{\theta },\hat{U})$ of their posterior distribution, assuming non‐informative priors.
3. Draw a new probability of observing x, ${\tilde{p}}_{r}$, from the normal approximation $N\left({\hat{p}}_{r},\frac{{\hat{p}}_{r}\left(1-{\hat{p}}_{r}\right)}{n}\right)$, where ${\hat{p}}_{r}$ is the sample proportion of the response indicator of x, ${\hat{p}}_{r}=\frac{n_{++}^{\text{obs}}}{n_{++}}$.
4. Draw a new probability of observed x = 1, ${\tilde{p}}_{x}$, from the normal approximation $N\left({\hat{p}}_{x},\frac{{\hat{p}}_{x}\left(1-{\hat{p}}_{x}\right)}{n}\right)$, where ${\hat{p}}_{x}$ is the observed proportion of x = 1, ${\hat{p}}_{x}=\frac{n_{1+}^{\text{obs}}}{n_{++}^{\text{obs}}}$.
5. Derive the value of the calibrated‐δ adjustment from the equation
  $$\frac{1}{n^{\text{mis}}}\sum_{i=1}^{n^{\text{mis}}}\text{expit}\left[\left({\tilde{\theta }}_{0}+\delta \right)+{\tilde{\theta }}_{y}y_{i}\right]=\frac{p_{x}^{\text{pop}}-{\tilde{p}}_{x}\;{\tilde{p}}_{r}}{1-{\tilde{p}}_{r}},$$
  where $p_{x}^{\text{pop}}$ is the probability of x = 1 in the population [Corrections added on 17 December 2018, after first online publication: the preceding equation has been corrected].
6. Fit the logistic regression imputation model for x conditional on y (in step 1) to the complete records with the intercept adjustment fixed to δ to obtain the maximum likelihood estimates of the imputation models' parameters $\hat{\theta }$ and their asymptotic sampling variance $\hat{U}$.
7. Draw new parameters $\dot{\theta }$ from the large‐sample normal approximation $N(\hat{\theta },\hat{U})$ of their posterior distribution, assuming non‐informative priors.
8. Draw imputed values for x from the aforementioned logistic regression imputation model, using the newly drawn parameters $\dot{\theta }$ and calibrated‐δ adjustment.

#### 3.1.2 Results

**Figure 1:**
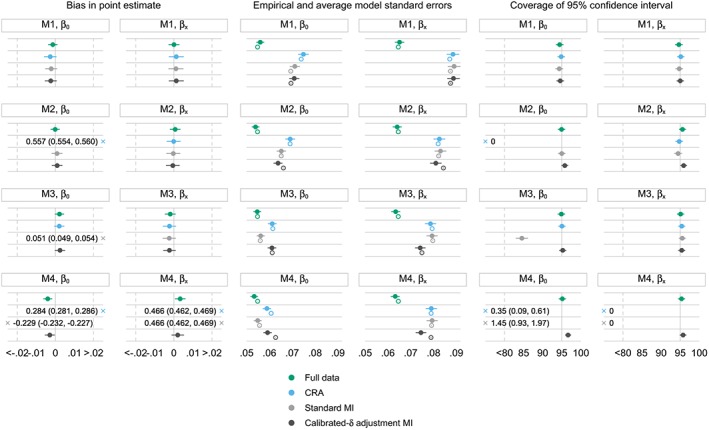
Simulation study: performance measures under different missingness mechanisms for x; β_0_= ‐0.693;β_x_ = 0.405. M1, x is missing completely at random; M2, x is missing at random conditional on y, M3, x is missing not at random dependent on x; M4, x is missing not at random dependent on x and y; error bars, ±1.95× Monte Carlo standard errors; filled and hollow points, empirical and average model standard errors, respectively; ×, out‐of‐range values. CRA, complete record analysis; MI, multiple imputation [Colour figure can be viewed at wileyonlinelibrary.com]

Results of the simulation study are summarised graphically in Figure 1. Full data and CRA both give the results that the theory predicts. Analysis of full data is always unbiased with coverage close to the 95% level and the smallest standard errors of all methods. The CRA is unbiased under M1 and M3 as expected,^28^ but bias is observed under the other two missingness mechanisms. Coverage is correspondingly low when bias is present, and efficiency is lower than that in full data.

Under M1, when *x* is MCAR, all methods appear unbiased, with comparable empirical and average model standard errors and correct coverage. This is as expected.

Under M2, when *x* is MAR conditional on *y*, CRA is severely biased in the estimate of *β*_0_ and the corresponding coverage of 95% CIs falls to 0. However, the method provides an unbiased estimate of *β*_*x*_ with correct coverage. This result is specific to this simulation set‐up, where the probability of being a complete record depends on the outcome, and the analysis model is a logistic regression. This mimics case‐control sampling, where the log odds of the logistic regression is biased in case‐control studies but the log OR is not.^28^, ^29^ The outcome‐covariate association can therefore be estimated consistently amongst the complete records. Standard MI and calibrated‐*δ* adjustment MI are unbiased for both parameter estimates. Standard MI yields comparable empirical and average model standard errors and coverage attains the nominal level. In calibrated‐*δ* adjustment MI, empirical standard errors are slightly smaller than the average model counterparts, leading to a minimal increase in coverage.

Under M3, when *x* is MNAR dependent on *x*, CRA yields unbiased estimates of both parameters. Standard MI is biased in the estimate of *β*_0_ but provides an unbiased estimate of *β*_*x*_ due to the symmetry property of the ORs. Generally, in the logistic regression with an incomplete covariate *x*, when the missingness mechanism is such that both standard MI and CRA are unbiased, standard MI tends not to be more efficient than CRA in estimating *β*_*x*_.^28^ This is because, without auxiliary variables in the imputation model, standard MI does not carry any extra information on the OR compared with CRA. This is seen in the simulation results for *β*_*x*_ under missingness mechanisms M1 to M3. Under M3, calibrated‐*δ* adjustment MI is also unbiased in both parameter estimates. Given that all three methods are unbiased for *β*_*x*_ under M3, there is a small gain in efficiency in the estimate of *β*_*x*_ in calibrated‐*δ* adjustment MI, as the empirical standard error for this parameter is slightly smaller than that in CRA. Under this missingness mechanism, empirical and average model standard errors are comparable across methods; for methods that are unbiased, their corresponding coverage of 95*%* CIs generally attains the nominal level.

Under M4, when *x* is MNAR dependent on *x* and *y*, standard MI and CRA are again biased in both parameter estimates, leading to coverage close or equal to 0. In contrast, calibrated‐*δ* adjustment MI produces unbiased estimates of both parameters. In this method, empirical standard errors are again slightly smaller than the average model counterparts (as seen previously under M2), which leads to coverage slightly exceeding the 95*%* level.

### 3.2 When the population distribution is estimated with uncertainty

So far, the population distribution of the incomplete covariate that is used to derive the calibrated‐*δ* adjustment is assumed to be obtained from a population census or equivalent. In other words, it is assumed that there is no uncertainty associated with estimating the reference distribution, and hence, the adjustment. In calibrated‐*δ* adjustment MI, we believe that the extra uncertainty in estimating the calibrated‐*δ* adjustment should be ignored when the population distribution of the incomplete covariate is assumed to be invariant, unless the reference population is not a census or equivalent. Since MI is a Bayesian procedure in which all sources of uncertainty are modelled, this explains why, if there is uncertainty about the population distribution of the incomplete covariate, this uncertainty needs to be accounted for in the derivation of the calibrated‐*δ* adjustment across imputations.

When the population distribution of the incomplete covariate is not “known” and is estimated, a natural approach for incorporating this extra uncertainty would be to draw values of the population proportions from their distribution and calculate the calibrated‐*δ* adjustment using these draws, so that this uncertainty is reflected in the MI variance estimation. This additional step is expected to have an effect on the between‐imputation variance of Rubin's variance estimator. An extension of the simulation study presented in Section 3.1 is conducted to explore this setting.

#### 3.2.1 Method

This extended simulation study of a fully observed binary outcome *y* and a partially observed binary covariate *x* follows the same method described in Section 3.1.1, except that two variations of the population proportions of *x* are evaluated in the imputation step of calibrated‐*δ* adjustment MI. The reference distribution is assumed to either come from a census or equivalent (case 1), or be estimated in an external data set of larger size (case 2) or smaller size (case 3) than the study sample.

Suppose that, in an external data set of size *n* ^ex^, which comes from the same population as the study sample, the sample proportion ${\hat{p}}_{x}^{\text{pop}}$ provides an unbiased estimate of the population proportion $p_{x}^{\text{pop}}$. Assuming that the sampling distribution of the sample proportions is approximately normal, its standard error is given by

$$\text{SE}\left({\hat{p}}_{x}^{\text{pop}}\right)=\sqrt{\frac{{\hat{p}}_{x}^{\text{pop}}\left(1-{\hat{p}}_{x}^{\text{pop}}\right)}{n^{\text{ex}}}}.$$

The data generating mechanism and analysis procedures are as follows.

1. For cases 2 and 3, the following two steps are performed to incorporate the sampling behaviour of ${\hat{p}}_{x}^{\text{pop}}$, which is estimated in an external data set of size *n* ^ex^, into the data generating mechanism in repeated simulations.
  a. Simulate *n* ^ex^ = 10 000 (case 2) or 1000 (case 3) complete values of the binary 0/1 covariate *x* from the model
    $$x∼\text{Bernoulli}\left(p_{x}^{\text{pop}}=0.7\right).$$
  b. Obtain the sample proportion ${\hat{p}}_{x}^{\text{pop}}$ of *x*, which is an unbiased estimate of the population proportion $p_{x}^{\text{pop}}$.
2. Simulate *n* = 5000 complete values of the binary 0/1 covariate *x* and binary 0/1 outcome *y* from the models
  (5) $$\begin{array}{ll} & x∼\text{Bernoulli}\left(p_{x}^{\text{pop}}=0.7\right);\\ & \text{logit}\left[p\left(y=1\;|\;x\right)\right]=\beta_{0}+\beta_{x}x, \end{array}$$
  where *β*_0_ and *β*_*x*_ are arbitrarily set to $\ln\left(0.5\right)$ and $\ln\left(1.5\right)$, respectively. The same values of the *β* coefficients are used throughout to make bias comparable across all simulation settings.
3. Simulate a binary indicator of response *r* of *x* from each of the selection models M1 to M4 (Table 1C). Values of 1.5 and −1.5 are chosen for *α*_*y*_ and *α*_*x*_ in M2 and M3, respectively. For M4, *α*_*y*_ = 1.5 and *α*_*x*_ = −1.5 are used. In all selection models, *α*_0_ is altered to achieve approximately 45*%* missing *x*. For M1, *α*_0_ is calculated directly as $\ln\left(\frac{0.55}{0.45}\right)$; for M2 to M4, *α*_0_ = −0.2; 1.35 and 0.75 are used.
4. For *i* = 1,…,5000, set *x*_*i*_ to missing if *r*_*i*_ = 0.
5. Impute missing values in *x* *M* = 20 times using standard MI and calibrated‐*δ* adjustment MI in turn. For cases 2 and 3, calibrated‐*δ* adjustment MI is performed as follows.
  a. Draw a value ${\tilde{p}}_{x}^{\text{pop}}$ from the normal approximation $N\left({\hat{p}}_{x}^{\text{pop}},\frac{{\hat{p}}_{x}^{\text{pop}}\left(1-{\hat{p}}_{x}^{\text{pop}}\right)}{n^{\text{ex}}}\right)$, with values of *n* ^ex^ = 10 000 (case 2) and 1000 (case 3). This is done by first taking a draw $\tilde{z}$ from the standard normal distribution, $z∼N\left(0,1\right)$, followed by drawing ${\tilde{p}}_{x}^{\text{pop}}={\hat{p}}_{x}^{\text{pop}}+\tilde{z}\sqrt{\frac{{\hat{p}}_{x}^{\text{pop}}\left(1-{\hat{p}}_{x}^{\text{pop}}\right)}{n^{\text{ex}}}}$ [Corrections added on 17 December 2018, after first online publication: the preceding equation has been corrected].
  b. Derive the calibrated‐*δ* adjustment and perform MI according to the algorithm set out in Section 3.1.1, using ${\tilde{p}}_{x}^{\text{pop}}$ as the reference proportion.
6. For each MI method, fit the analysis model (5) to each completed data set and combine the results using Rubin's rules.^1^, ^7^

Step 5 is designed to mimic the full Bayesian sampling process, which is always the aim in proper (or Rubin's) MI. Again, steps 1 to 6 are repeated *S* = 2000 times under each of the four selection models M1 to M4, so the same set of simulated independent data sets is used to compare the two MI methods under the same missingness scenario, but a different set of data sets is generated for each missingness scenario.^24^ The parameters of interest are *β*_0_ and *β*_*x*_. Bias in ${\hat{\beta }}_{0}$ and ${\hat{\beta }}_{x}$, efficiency in terms of the empirical and average model standard errors, and coverage of 95*%* CIs are calculated over 2000 repetitions for each combination of simulation settings,^25^ with analyses of full data and complete records also provided for comparison.

All simulations are performed in Stata 14^26^ with mi impute logit for standard MI, the community‐contributed command uvis logit 27 for calibrated‐*δ* adjustment MI, and mi estimate: logit for fitting the analysis model to the completed data sets and combining the results using Rubin's rules^1^, ^7^; simulated data sets are analysed using the community‐contributed command simsum.^25^

#### 3.2.2 Results

**Figure 2:**
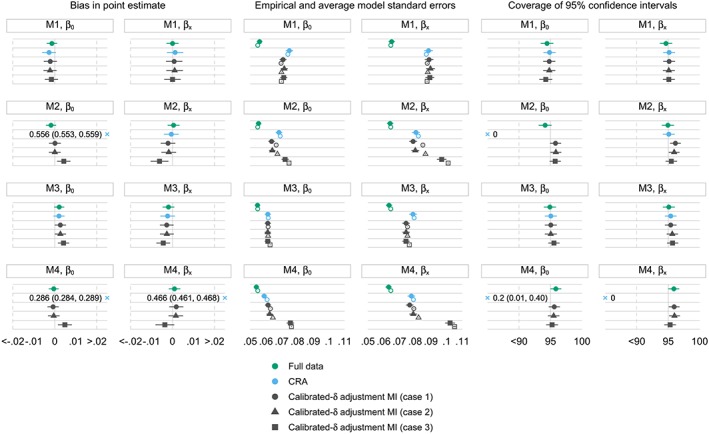
Extended simulation study: performance measures under different missingness mechanisms for x; β_0_= ‐0.693;β_x_ = 0.405; the population distribution of x is assumed to be invariant (case 1) or estimated in an external data set of size 10 000 (case 2) or 1000 (case 3). M1, x is missing completely at random; M2, x is missing at random conditional on y; M3, x is missing not at random dependent on x; M4, x is missing not at random dependent on x and y; error bars, ±1.95× Monte Carlo standard errors; filled and hollow points, empirical and average model standard errors, respectively; ×, out‐of‐range values. CRA, complete record analysis; MI, multiple imputation [Colour figure can be viewed at wileyonlinelibrary.com]

Results of the extended simulation study are presented in Figure 2. Bias in point estimates is similar when $p_{x}^{\text{pop}}$ is invariant or estimated in a large external data set (cases 1 and 2, respectively). Bias slightly increases, particularly under M2 and M4, when $p_{x}^{\text{pop}}$ is estimated in a small external data set with higher variance (case 3).

Empirical and average model standard errors are comparable and remain stable for calibrated‐*δ* adjustment MI across the three cases under M1 and M3. Under M2 and M4, the discrepancy previously seen between the empirical and average model standard errors in calibrated‐*δ* adjustment MI (Section 3.1.2) decreases in case 3 compared with cases 1 and 2. When there is increased uncertainty in estimating the population proportions of *x* (case 3 compared with case 1), there is also a marked increase in both the empirical and average model standard errors in calibrated‐*δ* adjustment MI. This extra uncertainty is reflected in the variation of the point estimates across the simulation repetitions according to how the simulation is set up, and is also acknowledged by an increase in the between‐imputation variance component of Rubin's variance estimator (Web Table A2).

In line with results seen for the standard errors, coverage attains the nominal level for calibrated‐*δ* adjustment MI under M1 and M3. Under M2 and M4, since the empirical standard errors are closer to the average model standard errors in case 3 compared with case 1, the slight over‐coverage of 95% CIs seen in case 1 seems to disappear in case 3.

## 4 CASE STUDY: ETHNICITY AND THE PREVALENCE OF TYPE 2 DIABETES DIAGNOSES IN THE HEALTH IMPROVEMENT NETWORK PRIMARY CARE DATABASE

This case study is conducted to illustrate the use of calibrated‐*δ* adjustment MI for handling missing data in ethnicity in UK primary care electronic health records, when ethnicity is included as a covariate in the analysis model. In particular, this is a cross‐sectional study, which examines the association between ethnicity and the prevalence of type 2 diabetes diagnoses in a large UK primary care database in 2013. Prevalence of type 2 diabetes is chosen as the outcome variable to illustrate the application of the calibrated‐*δ* adjustment MI method as developed and evaluated in Sections 2 and 3.

### 4.1 The Health Improvement Network database

The Health Improvement Network (THIN)^30^ is one of the largest databases in the UK to collect information on patient demographics, disease symptoms and diagnoses, and prescribed medications in primary care. THIN contains anonymised electronic health records from over 600 general practices across the UK, with more than 12 million patients contributing data. The database is broadly generalisable to the UK population in terms of demographics and crude prevalences of major health conditions.^31^, ^32^

Information is recorded during routine patient consultations with general practitioners from when the patients are registered with general practices participating in THIN to when they die or transfer out. Symptoms and diagnoses of disease are recorded using Read codes, a hierarchical coding system.^33^, ^34^ THIN also provides information on referrals made to secondary care and anonymised free text information. Patient demographics include information on year of birth, sex, and social deprivation status measured in quintiles of the Townsend deprivation score.^35^

The acceptable mortality reporting (AMR)^36^ and the acceptable computer usage (ACU)^37^ dates are jointly used for data quality assurance in THIN. The AMR date is the date after which the practice is deemed to be reporting a rate of all‐cause mortality sufficiently similar to that expected for a practise with the same demographics, based on data from the Office for National Statistics (ONS).^36^ The ACU date is designed to exclude the transition period between the practice switching from paper‐based records to complete computerisation; it is defined as the date from which the practice is consistently recording on average at least two drug prescriptions, ie, one medical record and one additional health record per patient per year.^37^

Use of THIN for scientific research was approved by the National Health Service South‐East Multicentre Research Ethics Committee in 2003. Scientific approval to undertake this study was obtained from IQVIA World Publications Scientific Review Committee in September 2017 (reference number 17THIN083).

### 4.2 Study sample

All individuals who are permanently registered with general practices in London contributing data to THIN are considered for inclusion in the study sample. This sample is chosen since it is not only more practical to perform MI on a smaller data set, but also because London is the most ethnically diverse region in the UK, and hence, incorrect assignment of ethnicity from imputing missing data with the White ethnic group is expected to be more apparent compared with other regions.

For each individual, a start date is defined as the latest of the following: date of birth, ACU and AMR dates,^36^, ^37^ and registration date. Similarly, an end date is defined as the earliest of the following: date of death, date of transfer out of practice, and date of last data collection from the practice. Point prevalence of type 2 diabetes on January 1, 2013 is calculated, since THIN is a dynamic database in which individuals start their registration with and leave their general practice at different times. Individuals are selected into the study sample if they are actively registered with practices in London on January 1, 2013, and in addition, they need to have been registered with the same practice for at least 12 months by this date. This criterion is introduced to ensure that there is enough time for the individuals to have their type 2 diabetes diagnoses recorded in their electronic health file after their registration with the practice.

### 4.3 Outcome variable and main covariate

The recording of diabetes diagnoses and management in THIN is comprehensive, and therefore, there are several ways an individual may be identified as diabetic. For this study, an algorithm developed by Sharma et al^38^ is used to identify individuals with diabetes mellitus, as well as to distinguish between type 1 and type 2 diabetes. According to this algorithm, individuals are identified as having diabetes if they have at least two of the following records: a diagnostic code for diabetes, supporting evidence of diabetes (eg, screening for diabetic retinophany), or prescribed treatment for diabetes. In this study, the first record of any of these three is considered as the date of diagnosis. In addition to identifying individuals with diabetes, the algorithm also distinguishes between type 1 and type 2 diabetes based on the individuals' age at diagnosis, types of treatment, and timing of the diabetes diagnosis.^38^, ^39^ After the study sample is selected using the method described in Section 4.2, prevalent cases of type 2 diabetes are defined as individuals who have a diagnosis of type 2 diabetes on or before January 1, 2013.

Ethnicity is typically recorded in THIN using the Read code system^33^; it can also be recorded using free text entries. A list containing Read codes related to ethnicity is developed using a published method.^34^ The majority of ethnicity records are identified by searching both the medical and additional health data files for Read codes in the ethnicity code list. Minimal additional information is found by searching the pre‐anonymised free text as well as other free text linked to ethnicity‐related Read codes. Ethnicity is then coded into the five‐level ONS classification as White, Mixed, Asian, Black, and Other ethnic groups.^40^ Subsequently, the Mixed and Other ethnic groups are combined due to the small counts and heterogeneity in these two groups. Searching for ethnicity‐related Read codes reveals that there is a small number of individuals with multiple inconsistent records of ethnicity. For these individuals, it cannot be determined with certainty whether their ethnicity is in fact one of the recorded categories or if all the recorded categories are incorrect. Therefore, their ethnicity is set to missing for simplicity, since the issue of inconsistency in ethnicity recording is not the focus of this study.

### 4.4 Statistical analysis

The analysis model in this study is a logistic regression model for a binary indicator of whether an individual has a diagnosis of type 2 diabetes on or before January 1, 2013, conditional on the individual's age in 2013, sex, Townsend deprivation score (five quintiles, from the least to the most deprived), and ethnic group (White, Asian, Black, and Mixed/Other). Age is analysed in 10‐year age groups for individuals aged 0 to 79 years, and all individuals aged 80 years and above are grouped into the 80+ category. Ethnicity information is extracted and categorised as described in Section 4.3. Since this study is conducted to illustrate the application of calibrated‐*δ* adjustment MI in a univariate missing data setting where missing data occurs in a single covariate (ethnicity), individuals with incomplete information on age, sex, and deprivation status were excluded from the analysis.

Missing values in ethnicity are handled by (i) a CRA, (ii) single imputation with the White ethnic group, (iii) standard MI, and (iv) calibrated‐*δ* adjustment MI using the 2011 ONS census distribution of ethnicity in London^40^ as the reference distribution. For MI of ethnicity, a multinomial logistic regression imputation model is constructed for ethnicity using all variables in the analysis model, including individuals' age group in 2013, sex, and quintiles of the Townsend score. In MI, the outcome variable must be explicitly included in the imputation model for the incomplete covariate.^2^ Since the analysis model is a logistic regression model, the binary indicator of type 2 diabetes is also included as a covariate in the imputation model for ethnicity.

In this study, ethnicity is analysed as a four‐level categorical variable. Therefore, the calibrated‐*δ* adjustment MI method for handling missing data in an incomplete binary covariate discussed in Sections 2 and 3 can be generalised for handling missing values in ethnicity as a categorical covariate. The overall proportion of the *j*th level of ethnicity, *j* = 1,…,4 can be written as

(6) $$p\left(\text{eth}=j\right)=p\left(\text{eth}=j\;|\;r=1\right)p\left(r=1\right)+p\left(\text{eth}=j\;|\;r=0\right)p\left(r=0\right),$$

where $p\left(\text{eth}=j\right)$ is available in the census; $p\left(\text{eth}=j\;|\;r=1\right)$, $p\left(r=1\right)$, and $p\left(r=0\right)$ can be obtained in the observed data.

A multinomial logistic regression imputation model for ethnicity, conditional on age group (40‐49 years old as the base level), sex (male as the base level), Townsend score (quintile 1 as the base level), and the binary indicator of type 2 diabetes (no diagnosis as the base level) is fitted to the observed data. Setting the first level of ethnicity (White, *j* = 1) as the base level to identify the model, the probability of the *j*th level of ethnicity in the observed data, *j* = 2,…,4 can be written in terms of the observed‐data linear predictors, ${\text{linpred}}_{j}^{\text{obs}}$, which is estimated from the multinomial logistic regression model for ethnicity as

(7) $$p\left(\text{eth}=j\;|\;r=1\right)=\frac{1}{n^{\text{obs}}}\sum_{i=1}^{n^{\text{obs}}}\frac{1}{1+\sum_{j=2}^{4}\;\text{exp}\left({\text{linpred}}_{ij}^{\text{obs}}\right)},$$

where *i* indexes individuals in the data set [Corrections added on 17 December 2018, after first online publication: the preceding equation has been corrected], and

(8) $$\begin{array}{ll} {\text{linpred}}_{ij}^{\text{obs}} & =\theta_{j0}^{\text{obs}}+\sum_{a=10}^{30}\theta_{j{\text{age}}_{a}}^{\text{obs}}I\left[{\text{age}}_{ij}=a\right]+\sum_{a=50}^{80}\theta_{j{\text{age}}_{a}}^{\text{obs}}I\left[{\text{age}}_{ij}=a\right]+\theta_{j\text{sex}}^{\text{obs}}I\left[{\text{sex}}_{ij}=\text{female}\right]\\ & \;+\sum_{t=2}^{5}\theta_{j{\text{town}}_{t}}^{\text{obs}}I\left[{\text{Townsend}}_{ij}=t\right]+\theta_{j\text{t2d}}^{\text{obs}}I\left[{\text{type 2 diabetes}}_{ij}=1\right]. \end{array}$$

Following the methods outlined in Section 3, since covariates in the imputation model for ethnicity are all binary or categorical, the relative risk ratios are the same amongst those with ethnicity observed and missing. The linear predictors in the missing data, ${\text{linpred}}_{j}^{\text{mis}}$, can therefore be written as

(9) $$\begin{array}{ll} {\text{linpred}}_{ij}^{\text{mis}} & =\left(\theta_{j0}^{\text{obs}}+\delta_{j0}\right)+\sum_{a=10}^{30}\theta_{j{\text{age}}_{a}}^{\text{obs}}I\left[{\text{age}}_{ij}=a\right]+\sum_{a=50}^{80}\theta_{j{\text{age}}_{a}}^{\text{obs}}I\left[{\text{age}}_{ij}=a\right]+\theta_{j\text{sex}}^{\text{obs}}I\left[{\text{sex}}_{ij}=\text{female}\right]\\ & \;+\sum_{t=2}^{5}\theta_{j{\text{town}}_{t}}^{\text{obs}}I\left[{\text{Townsend}}_{ij}=t\right]+\theta_{j\text{t2d}}^{\text{obs}}I\left[{\text{type 2 diabetes}}_{ij}=1\right], \end{array}$$

where *δ*_*j*0_ is the level‐*j* intercept adjustment in the multinomial logistic regression imputation model for ethnicity. Hence, the probability of the *j*th level of ethnicity in the missing data, *j* = 2,…,4, is given by

(10) $$p\left(\text{eth}=j\;|\;r=0\right)=\frac{1}{n^{\text{mis}}}\sum_{i=1}^{n^{\text{mis}}}\frac{1}{1+\sum_{j=2}^{4}\;\text{exp}\left({\text{linpred}}_{ij}^{\text{mis}}\right)}.$$

From (6), (7), (8), (9), (10) [Corrections added on 17 December 2018, after first online publication: Equation (10) has been corrected], to implement calibrated‐*δ* adjustment MI, we need to find the solutions *δ*_*j*0_, *j* = 2,…,4, to a system of three nonlinear equations for the three categories of ethnicity. The solutions to this system to equations can be obtained simultaneously using the Stata base command nl 26 and defining a function evaluator programme. Once the values of the calibrated‐*δ* adjustments are obtained, the imputation is performed using the same procedure as outlined in Section 3.1.

Both MI methods are performed using *M* = 30 imputations, and Rubin's rules^1^, ^7^ are used to obtain the final estimates of association and standard errors. All analyses are conducted using Stata 14,^26^ where mi impute mlogit is used for standard MI, the community‐contributed command uvis mlogit 27 for calibrated‐*δ* adjustment MI, and mi estimate: logit for performing the main analysis in the completed data sets and obtaining the final results using Rubin's rules.^1^, ^7^

### 4.5 Results

Of the $n=404\;318\left(3.0\%\right)$ individuals eligible for inclusion in the study sample (Web Figure A4), ethnicity is recorded for $309\;684\left(76.6\%\right)$ and missing for $94\;634\left(23.4\%\right)$ individuals (Web Table A3). Among individuals with ethnicity recorded, the estimated proportion of the White ethnic group is higher, and the non‐White ethnic groups lower, compared with the corresponding ethnic breakdown in the 2011 ONS census data for London. Single imputation with the White ethnic group and standard MI also overestimate the White group and underestimate the other non‐White groups, under the assumption that the ethnicity distribution in THIN should match that in the census. Calibrated‐*δ* adjustment MI imputes the majority of the missing ethnicity values with the Asian and Black groups and recovers the ethnic breakdown in the census as expected, since the census distribution is used as the reference (Web Table 3 and Web Figure A5).

**Figure 3:**
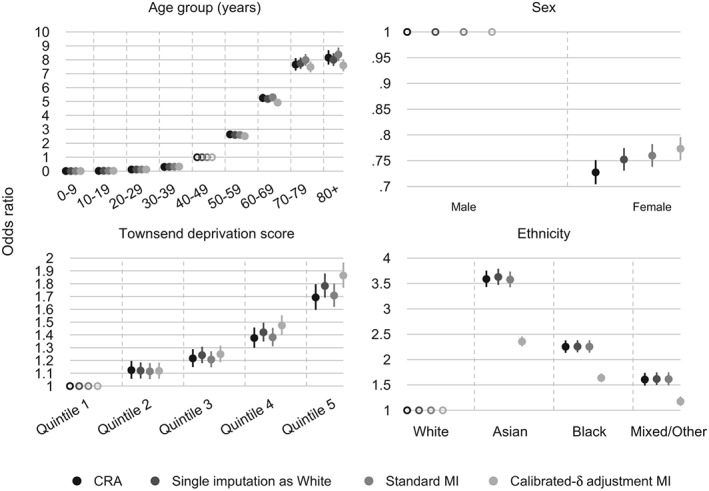
Case study: estimated odds ratio of having a type 2 diabetes diagnosis for age group (base level, 40 to 49 years), sex (base level, male), social deprivation status (base level, quintile 1 of the Townsend score), and ethnicity (base level, White) in different methods for handling missing ethnicity data; n = 404 318. Error bars, 95% confidence intervals. CRA, complete record analysis; MI, multiple imputation

Figure 3 and Web Table A4 present the estimated ORs of type 2 diabetes diagnosis and 95*%* CIs for age group, sex, Townsend score, and ethnicity in the analysis model. Age 40‐49 years, male, quintile 1, and the White ethnic group are selected as base levels for age group, sex, Townsend score, and ethnicity, respectively. *M* = 30 imputations produce Monte Carlo errors for point estimates of less than 10% of the estimated standard errors for all parameters. The relative efficiency versus an infinite number of imputations is above 0.988 for all parameter estimates and MI methods. Overall, the odds of being diagnosed with type 2 diabetes increase relatively smoothly with older age groups and higher quintiles of the Townsend score; are lower in women compared with men; and are higher in the Asian, Black, and Mixed/Other ethnic groups compared with the White group in all methods for handling missing data in ethnicity.

Compared with the other three methods under consideration, calibrated‐*δ* adjustment MI produces comparable estimated ORs for the younger age groups and smaller estimated ORs for the older (60+) age groups. Calibrated‐*δ* adjustment MI leads to a slightly higher estimated OR for women compared with CRA, single imputation with the White ethnic group, and standard MI; this increase is toward the null. All missing data methods produce ORs that increase with more deprived quintiles of the Townsend score. Calibrated‐*δ* adjustment MI yields similar estimated ORs compared with the other methods for the first three quintiles of the Townsend score and higher estimates for the top two quintiles.

The most noticeable differences in the point estimates associated with the prevalence of type 2 diabetes diagnoses are seen in the estimated ORs for ethnicity. Complete record analysis, single imputation with the White ethnic group, and standard MI again return similar results, in which the odds of having a diagnosis of type 2 diabetes are around 3.6 times higher in the Asian ethnic group compared with the White group, and individuals in the Black ethnic group are about 2.3 times more likely to receive a diagnosis compared with those of White ethnic background. Single imputation with the White ethnic group slightly increases the estimated ORs for the non‐White groups. This is because explanatory analyses conducted to examine predictors of both ethnicity and missingness in ethnicity suggest that individuals with missing ethnicity are, on average, less likely to have a diagnosis of type 2 diabetes (OR of observing ethnicity for type 2 diabetes diagnoses (adjusted for age group, sex, Townsend score) = 1.39, 95*%* CI 1.34 to 1.44). Replacing missing values with the White ethnic group means that this group will contain a lower percentage of type 2 diabetes diagnoses, which implies that the estimated ORs for the non‐White ethnic groups will increase. Compared with CRA, single imputation with the White ethnic group, and standard MI, calibrated‐*δ* adjustment MI leads to a reduction in the estimated ORs for the non‐White ethnic groups (Figure 3 and Web Table A4). For these groups, the 95*%* CIs of the point estimates for ethnicity in calibrated‐*δ* adjustment MI do not cross that of the other methods.

Fraction of missing information^11^ for the estimates of association between ethnicity and the prevalence of type 2 diabetes diagnoses was 0.132 (Monte Carlo standard error (MCSE) =0.003), 0.193 (MCSE =0.05), and 0.230 (MCSE =0.066) for the Asian, Black, and Mixed/Other ethnic groups, respectively, in standard MI. The corresponding quantities for these three groups in calibrated‐*δ* adjustment MI are 0.283 (MCSE =0.052), 0.245 (MCSE =0.045), and 0.327 (MCSE =0.051), respectively. Calibrated‐*δ* adjustment MI appears to have higher fraction of missing information estimates compared with standard MI. This could be explained by the fact that non‐White ethnic groups, which are underrepresented in the observed data, are imputed more often in calibrated‐*δ* adjustment MI than in standard MI. Therefore, the between‐imputation variance relies on more imputed values in these groups and less frequently imputed values in the White group, which leads to the non‐White proportion estimates being more variable across the completed data sets.

## 5 DISCUSSION

Our proposed calibrated‐*δ* adjustment MI method for missing data in a binary/categorical covariate involves utilising population‐level information about the incomplete covariate to generate a calibrated‐*δ* adjustment, which is then used in the intercept of the imputation model to improve the analysis of data generated by a MNAR mechanism. The development of this method was motivated by van Buuren et al's^13^ *δ* adjustment (offset) approach in MI, but where *δ* is derived based on external information instead of chosen arbitrarily or based on expert's belief (which is arguably not arbitrary, but can be subjective). Direct linkage to external data has also increasingly been used for the analysis of missing data suspected to be MNAR.^41^ However, externally linked data might not always be available, or the linkage might not be possible, whereas our proposed calibrated‐*δ* adjustment MI method does not require records from the same individuals to be directly linked between the data sets.

Under the MNAR assumption of missing data, MI results rely on subtle untestable assumptions, and may depend heavily on the particular way the missingness mechanism is modelled. This issue emphasises the central role of sensitivity analysis, which explores how inference may vary under different assumptions about the missingness mechanism.^42^ Multiple imputation offers flexibility for performing sensitivity analysis, since the imputation model can be tuned to incorporate possible departures from the MAR assumption.^11^, ^42^ Unfortunately, a sensitivity analysis is often not performed or reported sufficiently in practise,^43^, ^44^ a tendency abetted by the practical constraints of many applied projects. When the population‐level information about the incomplete covariate is available, our proposed calibrated‐*δ* adjustment MI method provides a useful tool for performing a single calibrated sensitivity analysis to assess the impact of potential departures from the MAR assumption.

The analytic study of a 2 × 2 contingency table with a binary outcome variable *y* and a binary covariate *x* gave insights into how the method works and will work for more general contingency table settings with one incomplete variable. The analytic study explored the appropriate derivation of the calibrated‐*δ* adjustment under increasingly complex missingness mechanisms. We showed that, when data in *x* were MNAR dependent on *x* or both *x* and *y*, appropriately adjusting the intercept of the imputation model sufficiently corrected bias in the analysis model's parameter estimates. Based on this setting, simulation studies were conducted to explore scenarios when the population distribution of *x* was either invariant (ie, “known”) or estimated in an external data set with uncertainty. Calibrated‐*δ* adjustment MI was shown to perform as well as standard MI in terms of bias when data were MAR. Furthermore, calibrated‐*δ* adjustment MI also produced unbiased parameter estimates with good coverage and was preferred to standard MI under the two general MNAR mechanisms being evaluated.

In the analytic and simulation studies, we did not consider the MNAR selection model where the probability of observing *x* depends on both *x*, *y*, and their interaction. We suspect that calibrated‐*δ* adjustment MI with a single intercept adjustment calculated based on the marginal distribution of *x* alone will not fully correct bias introduced by this missingness mechanism, and that an additional sensitivity parameter for the *x*‐*y* association is present. Information about the population distribution of *x* conditional on *y* might be required to produce unbiased estimates when the probability of observing *x* given *x* differs across the levels of *y*. However, such information might not always be available in practice. Similarly, when the outcome variable *y* is continuous, a second sensitivity parameter for the covariate‐outcome association in the imputation model is needed; we will explore this setting in another paper.

In the case study which examined the association between ethnicity and the prevalence of type 2 diabetes diagnoses in THIN, calibrated‐*δ* adjustment MI using information from census data yielded a more plausible estimate of the ethnicity distribution compared with CRA, single imputation of missing values with the White ethnic group, and standard MI. Subsequently, estimates of association for the non‐White ethnic groups produced by calibrated‐*δ* adjustment MI were lower than that in the other methods. In explanatory analyses, it was found that ethnicity was more likely to be recorded for individuals with a diagnosis of type 2 diabetes. By imputing missing values with the non‐White ethnic groups more frequently, calibrated‐*δ* adjustment MI led to a decrease in the percentage of prevalent type 2 diabetes cases amongst these groups, which we thought was the primary reason explaining the lower ORs compared with the other methods. In addition, it was also possible that the explanatory power of ethnicity for type 2 diabetes was partially diluted by the stronger effect of social deprivation status, which compensated for the reduction in the ORs for ethnicity. The ORs for Townsend deprivation score were higher in calibrated‐*δ* adjustment MI compared with CRA for the top two quintiles. These findings seemed to suggest that some effect of ethnicity was absorbed in Townsend score in calibrated‐*δ* adjustment MI, where deprivation status explained some of the effect which might otherwise have been explained by ethnicity. This could be attributed to a possibility that individuals of the Asian or Black ethnic background, whose ethnicity was not recorded, were more likely to belong to the more deprived quintiles of the Townsend score.

Given the missingness mechanisms considered thus far for the development of calibrated‐*δ* adjustment MI in Sections 2 and 3, results in the case study suggested a potential departure from the MAR assumption for missingness in ethnicity. This was because, conditional on the outcome variable and other fully observed variables included in the analysis model, standard MI did not yield a distribution of ethnicity that was comparable to the census ethnic breakdown. Ethnicity was also not likely to be MNAR dependent only on the values of ethnicity, since the point estimates in CRA and standard MI were broadly comparable. Results from the exploratory analyses examining the associations between covariates in the imputation model for ethnicity and missingness in ethnicity amongst the complete records suggested that age group, sex, Townsend score, and type 2 diabetes were factors likely to be associated with whether ethnicity was recorded. This finding indicated that ethnicity was likely to be MNAR dependent on the ethnic groups, fully observed outcome variable (type 2 diabetes diagnoses), as well as other fully observed covariates in the analysis model (age group, sex, and deprivation status).

The major strength of calibrated‐*δ* adjustment MI is its flexibility to be adapted to impute variables in a given data set whose distributions might be available in some external data. Here, we used census data for ethnicity in primary care electronic health records, but information obtained from other nationally representative data sets (such as the Health Survey for England^45^) could similarly be used to impute missing data in other health indicators routinely recorded in primary care, such as smoking status or alcohol consumption. In such instances, the variability associated with estimating the reference distribution used for calibration needs to be accounted for in calibrated‐*δ* adjustment MI as illustrated in Section 3.2; although, this source of uncertainty might be negligible depending on the size of the external data set.

Throughout this paper, we restricted our development of calibrated‐*δ* adjustment MI to the case of a single partially observed covariate. However, we believe this approach can be extended for handling missing data in more than one variable. Multivariate imputation by chained equations (MICE)^5^, ^13^ is a popular procedure for performing MI of multivariate missing data and is commonly implemented under the MAR assumption.^19^, ^20^ MICE is an iterative procedure, which requires the specification of an imputation model for each incomplete variable, conditional on all other variables. Our proposed univariate calibrated‐*δ* adjustment MI method can, in principle, be embedded into MICE to impute certain MNAR variables whose distributions are available externally, whilst the standard MI method can be used for the imputation of other variables assuming data are MAR. In the MICE approach, when there are several MNAR variables to be imputed, information from more than one external data source can potentially be drawn on and utilised in calibrated‐*δ* adjustment MI for these variables.

Finally, returning to the analytic and simulation studies, we did not consider the setting where both the outcome variable *y* and covariate *x* are incomplete. When *y* is MNAR dependent on its values and in addition to the population information on *x* we can obtain the marginal distribution of *y* from an external data set, then this information can be used in calibrated‐*δ* adjustment MI for *y* when *y* is imputed in the MICE algorithm. If *y* is MAR, then there must be some artificial mechanism whereby the data set is divided into two subsets, ie, one where *y* is MAR dependent on the observed values of *x* and another one where *x* is MNAR dependent on its values. In this setting, our proposed MI method should work for *x* when it is imputed in the MICE algorithm. The more complex missingness settings involving several incomplete covariates are subjected to ongoing work and will be reported in the future.

## Supporting information

SIM_8004‐Supp‐0001‐SIM_8004.pdf
