# Supplementary material for: Population‐calibrated multiple imputation for a binary/categorical covariate in categorical regression models
Source: Stat Med. 2018 Oct 16;38(5):792–808. doi: 10.1002/sim.8004 (PMC6492126; doi:10.1002/sim.8004)
Supplement: Supplementary file 1 — SIM_8004‐Supp‐0001‐SIM_8004.pdf [file SIM-38-792-s001.pdf]

Web Appendices

Population-calibrated multiple imputation for a binary/categorical covariate in categorical regression models

Tra My Pham[1], James R Carpenter[2,3], Tim P Morris[2], Angela M Wood[4], Irene Petersen[1]

[1]Department of Primary Care and Population Health, University College London, London, UK

[2]London Hub for Trials Methodology Research, MRC Clinical Trials Unit at UCL, London, UK

[3]Department of Medical Statistics, London School of Hygiene and Tropical Medicine, London, UK

[4]Department of Public Health and Primary Care, University of Cambridge, Cambridge, UK

A1 Analytic study – bias in a 2×2 contingency table

In this section, we show that when the incomplete covariate x is missing not at random (MNAR) dependent on x or both x and y, adjusting the intercept of the imputation model for x can sufficiently correct bias introduced by missing values in x.

A1.1 x is MNAR on x

When x is MNAR dependent on x (M3), the posited model for the response indicator r of x is given by

$$\text{logit}\left[p\left(r=1 \mid x\right)\right] = \alpha_0 + \alpha_x x, \tag{A1}$$

and the corresponding probabilities of observing x are

$$p\left(r=1 \mid x=j\right) = p_{r_j} = \text{expit}\left(\alpha_0 + \alpha_x x\right); \quad j = 0, 1.$$

Fitting the imputation model $\text{logit}\left[p\left(x=1 \mid y\right)\right] = \theta_0 + \theta_y y$ to subjects with observed and missing x results in the following log odds ratios

$$\begin{aligned}
\left[\theta_y \mid r=1\right] = \theta_y^{\text{obs}} &= \ln\left(\frac{n_{00}p_{r_0}n_{11}p_{r_1}}{n_{01}p_{r_0}n_{10}p_{r_1}}\right) = \ln\left(\frac{n_{00}n_{11}}{n_{01}n_{10}}\right); \\
\left[\theta_y \mid r=0\right] = \theta_y^{\text{mis}} &= \ln\left[\frac{n_{00}\left(1-p_{r_0}\right)n_{11}\left(1-p_{r_1}\right)}{n_{01}\left(1-p_{r_0}\right)n_{10}\left(1-p_{r_1}\right)}\right] = \ln\left(\frac{n_{00}n_{11}}{n_{01}n_{10}}\right),
\end{aligned}$$

respectively. Hence, $\theta_y^{\text{obs}} = \theta_y^{\text{mis}}$, which are also the same as the log odds ratio θ_y in the full data (i.e. before values in x are set to missing). The log odds of $x=1$ in the observed and missing data are given by

$$\begin{aligned}
\left[\theta_0 \mid r=1\right] = \theta_0^{\text{obs}} &= \ln\left(\frac{n_{10}p_{r_1}}{n_{00}p_{r_0}}\right); \\
\left[\theta_0 \mid r=0\right] = \theta_0^{\text{mis}} &= \ln\left[\frac{n_{10}\left(1-p_{r_1}\right)}{n_{00}\left(1-p_{r_0}\right)}\right],
\end{aligned}$$

respectively. This implies that the correct adjustment in the imputation model's intercept should be

$$\begin{aligned}\theta_0^{\text{mis}} - \theta_0^{\text{obs}} &= \ln\left[\frac{(1-p_{r_1})\,p_{r_0}}{(1-p_{r_0})\,p_{r_1}}\right] \\ &= \ln\left[\frac{\exp(\alpha_0)}{\exp(\alpha_0+\alpha_x)}\right] \\ &= -\alpha_x,\end{aligned}$$

which is minus the log odds ratio of observing x for $x = 1$ compared to $x = 0$ in (A1).

A1.2 x is MNAR dependent on x and y

When x is MNAR dependent on x and y (M4), the posited model for the response indicator r of x is given by

$$\text{logit}\left[p\left(r=1 \mid x,y\right)\right] = \alpha_0 + \alpha_x x + \alpha_y y, \tag{A2}$$

and the corresponding probabilities of observing x are

$$p\left(r=1 \mid x=j, y=k\right) = p_{r_{jk}} = \text{expit}\left(\alpha_0 + \alpha_x x + \alpha_y y\right); \quad j,k = 0,1.$$

Fitting the imputation model logit $[p\,(x = 1 \mid y)] = \theta_0 + \theta_y y$ to subjects with observed and missing x results in the following log odds ratios

$$\theta_y^{\text{obs}} = \ln\left(\frac{n_{00}p_{r_{00}}n_{11}p_{r_{11}}}{n_{01}p_{r_{01}}n_{10}p_{r_{10}}}\right); \tag{A3}$$

$$\theta_y^{\text{mis}} = \ln\left[\frac{n_{00}\left(1-p_{r_{00}}\right)n_{11}\left(1-p_{r_{11}}\right)}{n_{01}\left(1-p_{r_{01}}\right)n_{10}\left(1-p_{r_{10}}\right)}\right]. \tag{A4}$$

Again, it can be shown from (A3) and (A4) that $\theta_y^{\text{obs}} = \theta_y^{\text{mis}}$, since

$$\begin{aligned}\theta_y^{\text{mis}} - \theta_y^{\text{obs}} &= \ln\left[\frac{(1-p_{r_{00}})(1-p_{r_{11}})\,p_{r_{01}}p_{r_{10}}}{(1-p_{r_{01}})(1-p_{r_{10}})\,p_{r_{00}}p_{r_{11}}}\right] \\ &= \ln\left[\frac{\exp(\alpha_0+\alpha_x)\exp(\alpha_0+\alpha_y)}{\exp(\alpha_0)\exp(\alpha_0+\alpha_x+\alpha_y)}\right] \\ &= 0.\end{aligned}$$

The log odds of $x = 1$ in the observed and missing data are given by

$$\theta_0^{\text{obs}} = \ln\left(\frac{n_{10}p_{r_{10}}}{n_{00}p_{r_{00}}}\right);$$

$$\theta_0^{\text{mis}} = \ln\left[\frac{n_{10}\left(1-p_{r_{10}}\right)}{n_{00}\left(1-p_{r_{00}}\right)}\right],$$

implying that the correct adjustment in the imputation model's intercept should be

$$\begin{aligned}\theta_0^{\text{mis}} - \theta_0^{\text{obs}} &= \ln\left[\frac{(1-p_{r_{10}})\,p_{r_{00}}}{(1-p_{r_{00}})\,p_{r_{10}}}\right] \\ &= \ln\left[\frac{\exp(\alpha_0)}{\exp(\alpha_0+\alpha_x)}\right] \\ &= -\alpha_x,\end{aligned}$$

which is again minus the log odds ratio of observing x in (A2).

A2 Weighted multiple imputation for a binary/categorical covariate

The procedure of the weighted multiple imputation (MI) method is as follows. In the imputation step, weights derived from the population marginal distribution of the incomplete variable are attached to the complete records, and a weighted (multinomial) logistic regression model is fitted to the complete records to obtain the maximum likelihood estimates of the imputation model's parameters $\widehat{\boldsymbol{\theta}}$ and their asymptotic sampling variance $\widehat{\boldsymbol{U}}$. New parameters are then drawn from the large-sample normal approximation $N(\widehat{\boldsymbol{\theta}}, \widehat{\boldsymbol{U}})$ of its posterior distribution, assuming non-informative priors. Finally, imputed values are drawn from the (multinomial) logistic regression using these new parameters. Note that *no weights* are used when fitting the substantive scientific model to the imputed data.

A2.1 Derivation of the marginal weights

The idea of augmenting the standard MI method with weights is related to the technique of post-stratification weighting, which is commonly used in survey non-responses when the population distributions are known.[1] To post-stratify the sample, weights are calculated to bring the sample distribution in line with the population. Suppose that in a survey, one of the variables measured is ethnicity, which is categorised into four groups (White, Black, Asian, and Mixed/Other). If the population distribution of ethnicity is available, the distribution of ethnicity among survey respondents can be compared with the population distribution. Suppose that a proportion $p^{\text{obs}} = 0.8$ of the survey respondents give their ethnicity as White, whereas the population has $p^{\text{pop}} = 0.6$ in this category. The White category is over-represented in the survey respondents, but can be made representative of the population by assigning to the responses a post-stratification weight $w^{\text{ps}} < 1$, such that

$$w^{\text{ps}} = 1/(p^{\text{obs}}/p^{\text{pop}}) = 1/(0.8/0.6) = 0.75.$$

In adapting this idea to MI, we need to address the complication arising because the *completed* data obtained after MI consist of both observed and imputed (missing) data. Naive use of post-stratification weights in MI will recover the correct population distribution in the imputed data. However, since the observed data remain the same, the distribution in the completed data will not be matched to that in the population. Therefore, some *compensation* for the lack of representativeness in the observed data is needed in the imputed data so that the correct population distribution can be recovered after imputation. Continuing with the survey example, suppose that we survey 200 individuals, 100 of whom respond with their ethnicity. A proportion $p^{\text{obs}} = 0.8$ of these 100 responses are in the White group. If the population proportion of this group is $p^{\text{pop}} = 0.6$, we would expect to have 120 White individuals in the survey sample. This implies that among the 100 individuals with missing ethnicity, we need to impute ethnicity of 40 individuals as White, i.e. the proportion of the White category required in the missing data, p^{req}, is equal to 0.4. To make the completed (observed and imputed) data of this category representative of the population, we need to weight respondents of this category in the imputation model by

$$1/(p^{\text{obs}}/p^{\text{req}}) = 1/(0.8/0.4) = 0.5,$$

which is smaller than the corresponding naive post-stratification weight above, since it compensates for the over-representation among the survey respondents of White ethnicity.

More generally, suppose that we seek to collect a J-level variable x in a sample of size n, resulting in x being observed for n^{obs} subjects and missing for n^{mis} subjects, $n^{\text{obs}} + n^{\text{mis}} = n$. Let p_j^{obs} and p_j^{req} denote the level-j

[1]Raghunathan T. *Missing data analysis in practice.* Boca Raton: Chapman & Hall/CRC; 2015.

proportions of x in the observed and imputed data respectively, such that $p_j^{\text{obs}} n^{\text{obs}} = n_j^{\text{obs}}$, and $p_j^{\text{req}} n^{\text{mis}} = n_j^{\text{req}}$, where $j = 1, \ldots, J$. Let p_j^{pop} denote the level-j proportion of x in the population, which is assumed to be known. The aim here is to find p_j^{req} for each level of x such that the number of subjects in the completed data after imputation is equal to the expected number implied by the corresponding population proportion, i.e. $n_j^{\text{obs}} + n_j^{\text{req}} = p_j^{\text{pop}} n$. The level-$j$ proportion of x required in the imputed data, p_j^{req}, is given by

$$p_j^{\text{req}} = \frac{p_j^{\text{pop}} n - p_j^{\text{obs}} n^{\text{obs}}}{n^{\text{mis}}}.$$

Therefore, the weight for group j, which we refer to as the 'marginal weight' and denote by w_j^{m}, is

$$w_j^{\text{m}} = 1/(p_j^{\text{obs}} / p_j^{\text{req}}).$$

A2.2 Derivation of the conditional weights

The marginal weights introduced above only depend on the population distribution of the incomplete variable. However, if there are (fully observed) covariates in the imputation model, the associations between these variables and the incomplete variable distribution are not reflected in such weights. We therefore adjust the marginal weights to obtain another set of weights, termed the 'conditional weights', which account for covariates in the imputation model. These weights are derived using the marginal distribution of the incomplete variable obtained after having estimated the parameters of an imputation model assuming missing at random (MAR) in the complete records. Suppose that an imputation model is fitted to the complete records, and the corresponding predicted probabilities of the incomplete variable (averaged over the covariates) are obtained and applied to the missing data. Let p_j^{pred} denote the resulting predicted level-j proportion of x in the completed data, then the level-j proportion required in the imputed data is given by

$$p_j^{\text{req}} = \frac{p_j^{\text{pop}} n - p_j^{\text{pred}} n^{\text{obs}}}{n^{\text{mis}}},$$

and the conditional weight for group j, denoted by w_j^{c} , is

$$w_j^{\text{c}} = 1/(p_j^{\text{pred}} / p_j^{\text{req}}).$$

In this approach, the effects of covariates in the imputation model are reflected in the predicted probabilities p_j^{pred}, which are then used to derive the conditional weights for weighted MI.

A2.3 Analytic study – bias in a 2×2 contingency table

In a 2×2 contingency table of a complete binary outcome variable y and an incomplete binary covariate x (Section 2), we calculate analytic bias in the analysis model's parameter estimates (defined as $\hat{\beta} - \beta$) after missing values in x are handled by (i) a complete record analysis (CRA), (ii) standard MI, (iii) marginal weighted MI, and (iv) conditional weighted MI. The analytic calculations are then verified by simulating a full-data sample with $n = 10\,000$ observations of x and y from the following model

$$x \sim \text{Bernoulli}\,(p_x^{\text{pop}} = 0.7)\,;$$
$$\text{logit}\,[p\,(y = 1 \mid x)] = \beta_0 + \beta_x x,$$

where $\beta_0 = \ln(0.5)$ and $\beta_x = \ln(1.5)$. Missing values in x are generated using selection models M1–M4 with a range of values for the selection parameters αs (Table A1).

Figures A1–A3 present the analytic bias in CRA, standard MI, marginal and conditional weighted MI under MAR and MNAR mechanisms with various values of the selection parameters. When x is missing completely at

Table A1 Analytic study: values of selection parameters for generating missingness in x used in simulations conducted to verify analytic calculations. r: response indicator of x.

Missingness model	Linear predictor of selection model logit $[p[(r = 1 \mid x, y)]$	Selection parameter			% missing x
		α_0	α_x	α_y	
M1	α_0	$[-3, 3]$			5–95
M2	$\alpha_0 + \alpha_y y$	$[-3, 3]$		$[-3, 3]$	3–97
M3	$\alpha_0 + \alpha_x x$	$[-3, 3]$	$[-3, 3]$		2–98
M4	$\alpha_0 + \alpha_x x + \alpha_y y$	0.5	$[-3, 3]$	$[-3, 3]$	9–84

random (MCAR) (M1), all methods provide unbiased parameter estimates, as suggested by the analytic calculations (results not shown).

When x is MAR conditional on y (M2, Figure A1), standard MI and conditional weighted MI are unbiased, while bias is observed for CRA in β_0, and for marginal weighted MI in both parameter estimates. This bias is due to the marginal weights not accounting for the association between x and y in the imputation model for x. As a result, marginal weights do not successfully recover the correct distribution of x after MI.

Both parameter estimates are unbiased in marginal weighted MI when x is MNAR dependent on x (M3, Figure A2), while standard MI leads to noticeable bias in the estimate of β_0. Bias in conditional weighted MI is small and occurs for extreme values of the selection parameters. Since missingness in x does not depend on y under M3, CRA is unbiased in both parameter estimates as the theory predicts.

Under the last missingness mechanism when x is MNAR dependent on both x and y (M4, Figure A3), none of the methods result in unbiased parameter estimates. However, bias appears to be the smallest in conditional weighted MI. Although bias is present in both standard MI and marginal weighted MI, the magnitude of bias is smaller in marginal weighted MI compared to standard MI. Under this missingness mechanism, conditional weighted MI can be regarded as a hybrid of marginal weighted MI and standard MI. The conditional weights correct for some bias introduced by x in the selection model in a similar manner to the marginal weights under M3; the method also alleviates some residual bias similarly to standard MI under M2.

Overall, these results suggest that under the missingness mechanisms considered in this paper, calibrated-δ adjustment MI provides a more general solution for accommodating missing data in x and is therefore the preferred method compared to standard MI and (marginal and conditional) weighted MI.

Figure A1 Analytic study: analytic bias when x is MAR conditional on y (M2). Selection parameters $\alpha_0 \in [-3, 3]$, $\alpha_y \in [-3, 3]$; corresponding percentages of missing x are presented for extreme values (± 3) of the α parameters.

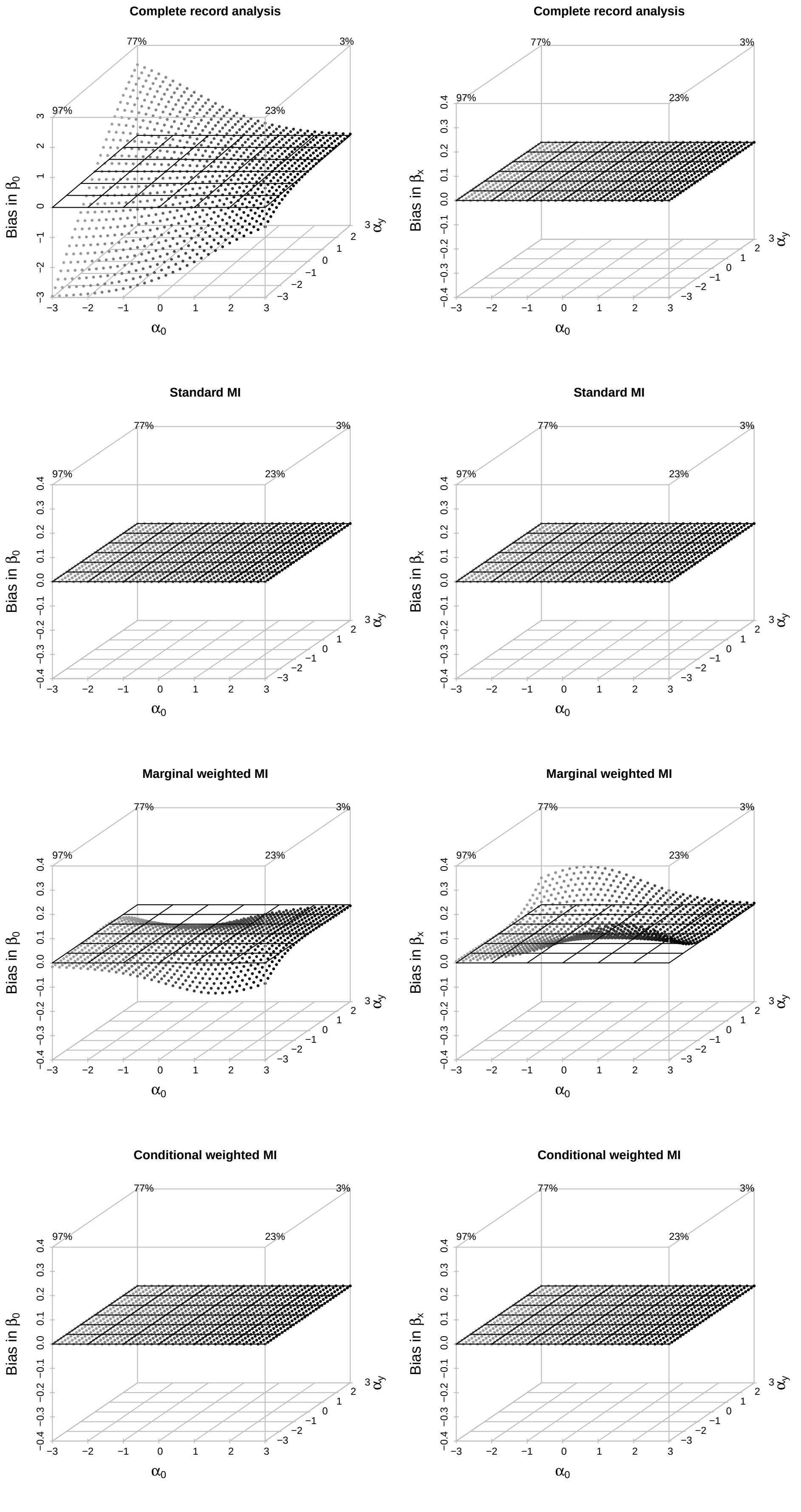

Figure A2 Analytic study: analytic bias when x is MNAR dependent on x (M3). Selection parameters $\alpha_0 \in [-3, 3]$, $\alpha_x \in [-3, 3]$; corresponding percentages of missing x are presented for extreme values (± 3) of the α parameters.

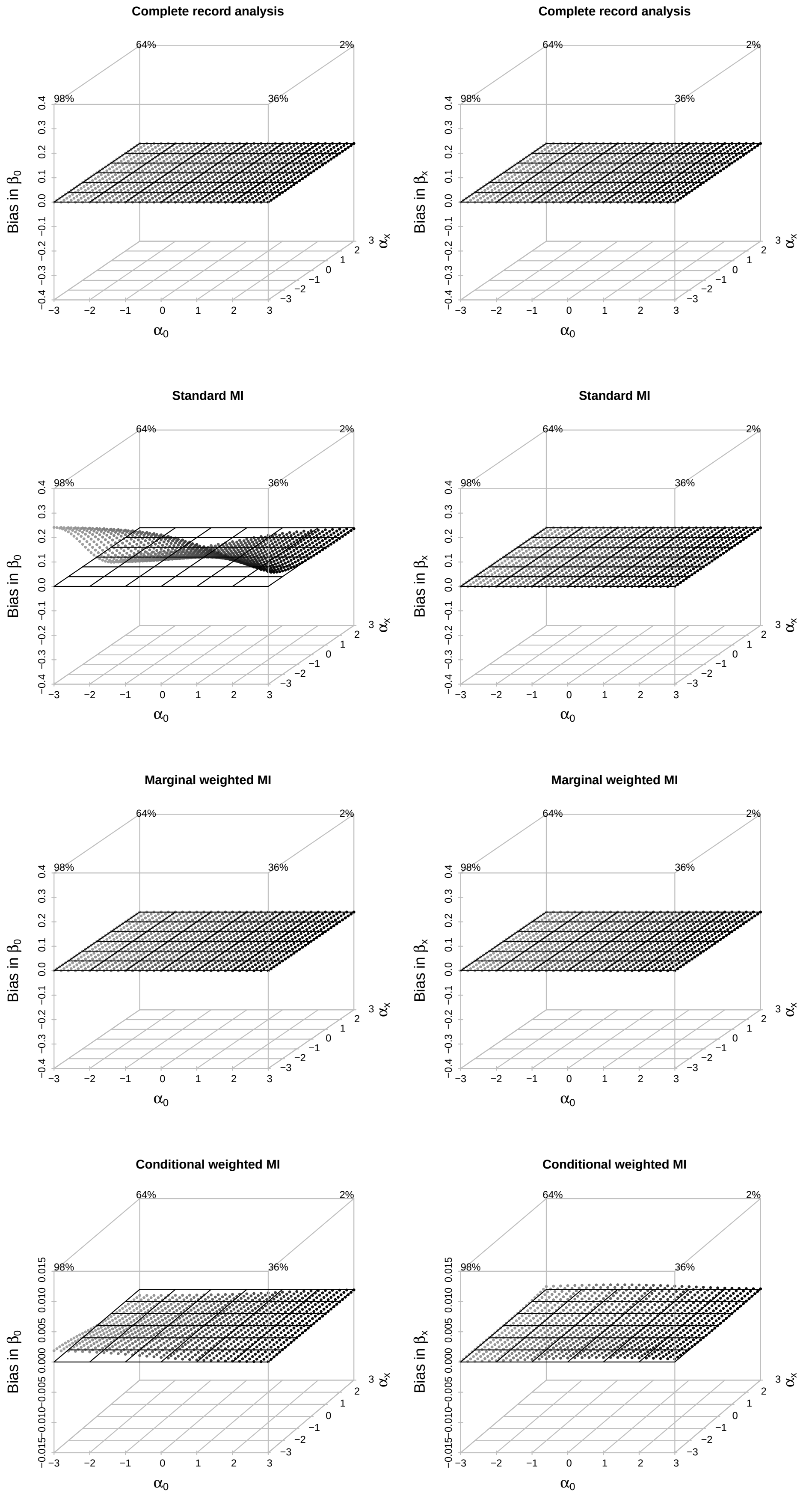

Figure A3 Analytic study: analytic bias when x is MNAR dependent on x and y (M4). Selection parameters $\alpha_0 = 0.5, \alpha_x \in [-3, 3]$, $\alpha_y \in [-3, 3]$; corresponding percentages of missing x are presented for extreme values (± 3) of the α parameters.

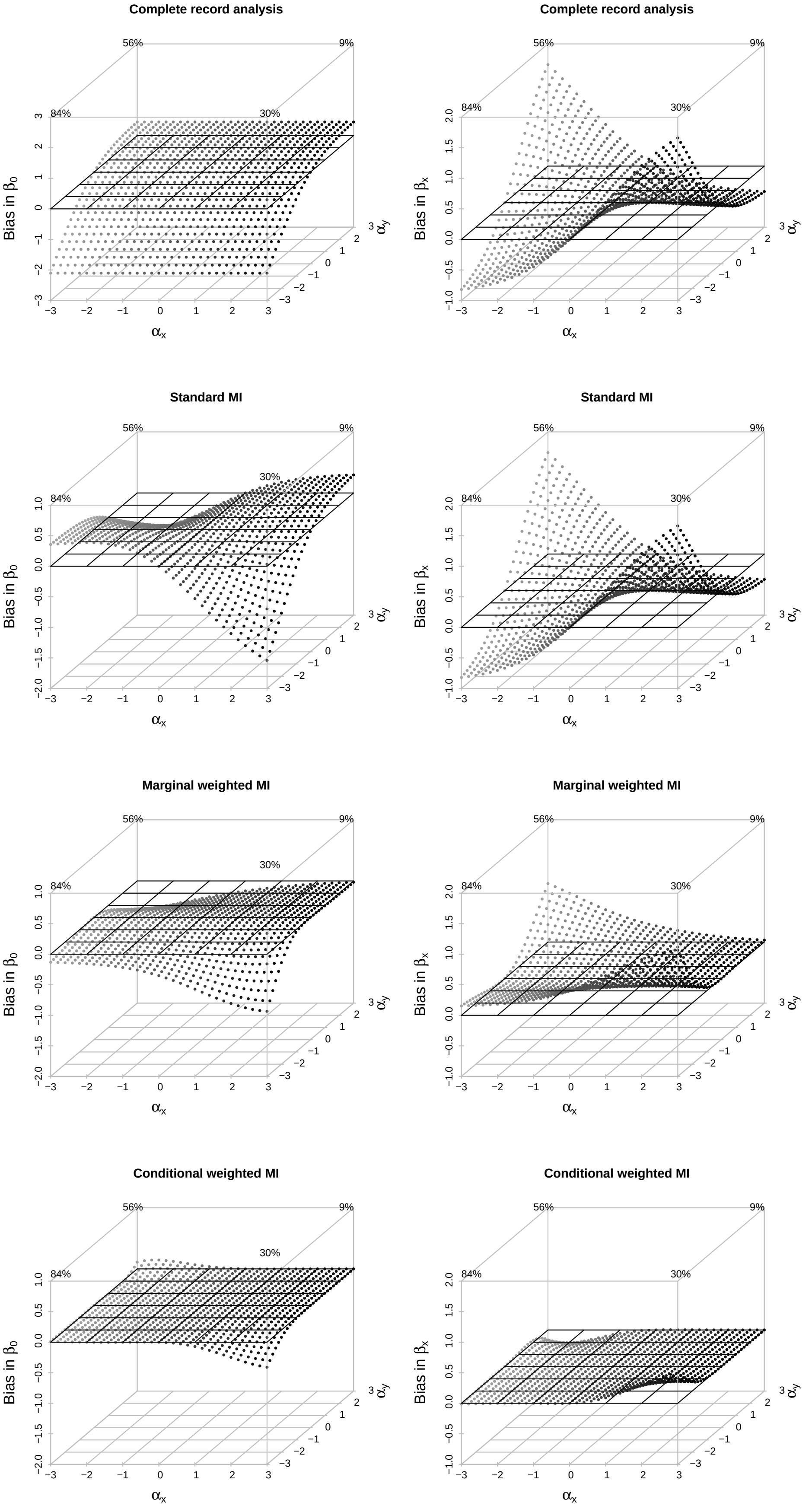

A3 Simulation studies: when the population distribution is estimated with uncertainty

Table A2 Extended univariate simulation study: variance information about the β parameter estimates in calibrated-δ adjustment MI in one simulation repetition, when x is MNAR dependent on x and y (M4); the population distribution of x is assumed to be invariant (case 1) or estimated in an external dataset of size 10 000 (case 2) or 1 000 (case 3). $\widehat{W}$, within-imputation variance; $\widehat{B}$, between-imputation variance; Total, total variance; RVI, relative increase in variance; FMI, fraction of missing information; RE, relative efficiency; MCSE, Monte Carlo standard error.

(a) Case 1

	$\widehat{W}$	$\widehat{B}$	Total	RVI	FMI	RE
$\hat{\beta}_0$	0.0030	0.0008	0.0039	0.2773	0.2210	0.9891
MCSE	<0.0001	0.0002	0.0002	0.0689	0.0442	0.0023
$\hat{\beta}_x$	0.0042	0.0018	0.0061	0.4488	0.3167	0.9844
MCSE	<0.0001	0.0004	0.0005	0.1121	0.0563	0.0029

(b) Case 2

	$\widehat{W}$	$\widehat{B}$	Total	RVI	FMI	RE
$\hat{\beta}_0$	0.0030	0.0007	0.0038	0.2579	0.2085	0.9897
MCSE	<0.0001	0.0002	0.0002	0.0650	0.0430	0.0022
$\hat{\beta}_x$	0.0041	0.0016	0.0059	0.4111	0.2976	0.9853
MCSE	<0.0001	0.0004	0.0004	0.1066	0.0569	0.0029

(c) Case 3

	$\widehat{W}$	$\widehat{B}$	Total	RVI	FMI	RE
$\hat{\beta}_0$	0.0031	0.0030	0.0062	1.0038	0.5136	0.9750
MCSE	<0.0001	0.0009	0.0009	0.2899	0.0782	0.0039
$\hat{\beta}_x$	0.0042	0.0067	0.0113	1.6558	0.6380	0.9691
MCSE	<0.0001	0.0019	0.0020	0.4645	0.0708	0.0035

A4 Case study – ethnicity and the prevalence of type 2 diabetes diagnoses in The Health Improvement Network primary care database

Figure A4 Case study: flowchart of selection criteria for the study sample taken from The Health Improvement Network database. An individual can be excluded from the study sample due to more than one criterion.

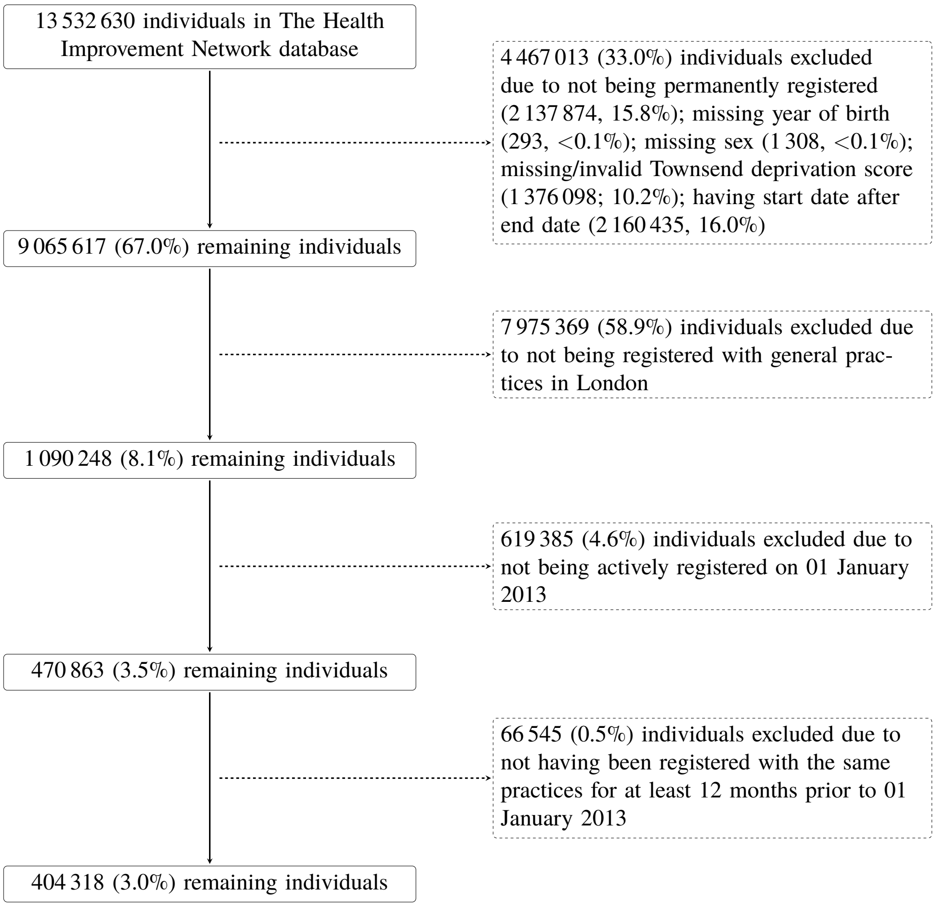

Table A3 Case study: summary of variables in the analysis; $n = 404\,318$.

(a) Distribution of age group, sex, Townsend deprivation score, and type 2 diabetes diagnoses.

Variable	Frequency	%
Age group (years)		
0–9	41 601	10.29
10–19	45 664	11.29
20–29	50 065	12.38
30–39	65 695	16.25
40–49	64 837	16.04
50–59	53 272	13.18
60–69	39 427	9.75
70–79	25 348	6.27
80+	18 409	4.55
Sex		
Male	198 301	49.05
Female	206 017	50.95
Townsend score		
Quintile 1 (least deprived)	48 934	12.10
Quintile 2	64 788	16.02
Quintile 3	101 305	25.06
Quintile 4	102 626	25.38
Quintile 5 (most deprived)	86 665	21.43
Type 2 diabetes	22 100	5.47

(b) Distribution of ethnicity when missing values are (i) included; (ii) excluded; (iii) and imputed with the White ethnic group.

Ethnicity	Frequency	% including missing	% excluding missing	Frequency missing imputed with White	% missing imputed with White	% 2011 Office for National Statistics census London
White	224 403	55.50	72.46	319 037	78.91	59.8
Asian	35 027	8.66	11.31	35 027	8.66	18.8
Black	30 771	7.61	9.94	30 771	7.61	13.3
Mixed/Other	19 483	4.82	6.29	19 483	4.82	8.4
Missing	94 634	23.41				
$\sum$ including missing	404 318					
$\sum$ excluding missing	309 684					

Figure A5 Case study: distribution of four-level ethnicity from different methods for handling missing data in ethnicity, in comparison with the 2011 Office for National Statistics census distribution in London (represented by horizontal black lines); $n = 404\,318$. 95% confidence intervals were very narrow and therefore omitted.

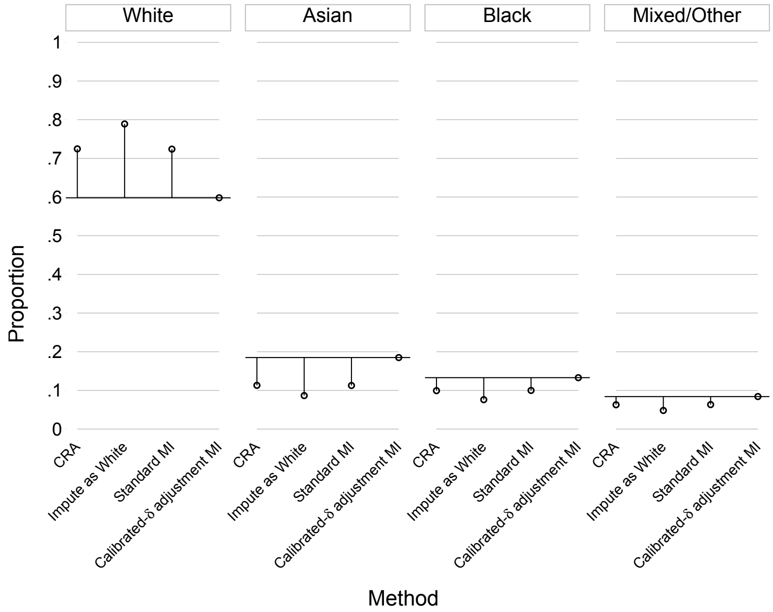

Table A4 Case study: adjusted oods ratio and 95% confidence interval from a multivariable logistic regression model for the prevalence of type 2 diabetes diagnoses, conditional on age group in 2013, sex, Townsend deprivation score, and ethnic group in different methods for handling missing data in ethnicity; $n = 404\,138$. CI, confidence interval.

	CRA		Single imputation with White		Standard MI		Calibrated-δ adjustment MI	
	Odds ratio	95% CI	Odds ratio	95% CI	Odds ratio	95% CI	Odds ratio	95% CI
Age group (years)								
0–9	0.010	0.006 to 0.016	0.010	0.006 to 0.016	0.010	0.006 to 0.016	0.010	0.006 to 0.017
10–19	0.022	0.016 to 0.032	0.026	0.020 to 0.035	0.025	0.019 to 0.033	0.025	0.019 to 0.033
20–29	0.120	0.103 to 0.139	0.122	0.107 to 0.139	0.120	0.106 to 0.137	0.122	0.107 to 0.139
30–39	0.308	0.283 to 0.336	0.316	0.292 to 0.342	0.320	0.296 to 0.347	0.330	0.305 to 0.357
40–49	1		1		1		1	
50–59	2.641	2.495 to 2.796	2.605	2.474 to 2.743	2.604	2.473 to 2.742	2.516	2.390 to 2.649
60–69	5.255	4.968 to 5.559	5.190	4.933 to 5.46	5.309	5.044 to 5.587	4.928	4.685 to 5.184
70–79	7.662	7.230 to 8.120	7.748	7.352 to 8.166	7.984	7.573 to 8.417	7.484	7.102 to 7.886
80+	8.154	7.655 to 8.685	8.003	7.560 to 8.472	8.379	7.910 to 8.876	7.596	7.175 to 8.043
Sex								
Male	1		1		1		1	
Female	0.727	0.704 to 0.751	0.752	0.731 to 0.774	0.760	0.738 to 0.782	0.773	0.751 to 0.796
Townsend score								
Quintile 1 (least deprived)	1		1		1		1	
Quintile 2	1.125	1.057 to 1.196	1.121	1.060 to 1.185	1.115	1.054 to 1.179	1.119	1.058 to 1.183
Quintile 3	1.217	1.149 to 1.288	1.242	1.180 to 1.307	1.208	1.147 to 1.272	1.249	1.187 to 1.316
Quintile 4	1.376	1.300 to 1.457	1.420	1.349 to 1.496	1.381	1.312 to 1.455	1.474	1.400 to 1.553
Quintile 5 (most deprived)	1.693	1.596 to 1.796	1.783	1.691 to 1.879	1.708	1.619 to 1.802	1.864	1.768 to 1.966
Ethnicity								
White	1		1		1		1	
Asian	3.588	3.431 to 3.753	3.629	3.474 to 3.789	3.577	3.425 to 3.735	2.355	2.259 to 2.456
Black	2.253	2.135 to 2.378	2.257	2.142 to 2.379	2.254	2.136 to 2.379	1.638	1.555 to 1.725
Mixed/Other	1.606	1.486 to 1.736	1.617	1.497 to 1.746	1.615	1.491 to 1.749	1.174	1.085 to 1.270
